# *Baylisascaris procyonis* (Chromadorea Ascarididae): Case Study of the Little-Known Human Health Threat That Is Literally in Your Backyard

**DOI:** 10.3390/tropicalmed10060156

**Published:** 2025-06-02

**Authors:** Scott E. Henke

**Affiliations:** Caesar Kleberg Wildlife Research Institute, Texas A&M University-Kingsville, MSC 218, 1150 Engineering Avenue, Kingsville, TX 78363, USA; scott.henke@tamuk.edu

**Keywords:** *Baylisascariasis*, *Baylisascaris procyonis*, neural larva migrans, *Procyon lotor*, raccoon, zoonotic parasite

## Abstract

*Baylisascariasis* is a debilitating and potentially lethal zoonotic disease caused by a nematode parasite that has a worldwide distribution. *Baylisascaris* spp. are carried by a variety of mammalian definitive hosts, and their larvae can infect a large diversity of paratenic hosts including birds and mammals, and even humans. Herein, the potential exposure risk of this zoonotic parasite is demonstrated through the study of a suburban American community with a population of *Baylisascaris procyonis*—infected raccoons (*Procyon lotor*) as a case study for any location with *Baylisascaris* spp., definitive hosts, and proximity to humans. Soil from 100 properties within neighborhoods of southern Corpus Christi, TX, USA, was surveyed to determine if viable *B. procyonis* eggs were present. In total, 27% of the residential properties were contaminated. Positive soil samples, on average, contained 31,287 *B. procyonis* eggs/gram of soil; of these samples, 92% of the *B. procyonis* eggs had motile larvae. Sites with contaminated soils appeared random within residential properties; frequency of contaminated sites was similar between known raccoon defecation sites and random sites. Suggestions for the reduction in risks of exposure to this potentially debilitating parasite are offered to residents of *Baylisascaris*-contaminated properties.

## 1. Introduction

*Baylisascariasis* is a zoonotic, parasitic disease potentially caused by all ten species of intestinal ascarids of the genus *Baylisascaris* Sprent 1968 [1,2,3,4]. The roundworms of the genus *Baylisascaris* have a cosmopolitan distribution, occurring throughout much of North America, South America, Europe, and Asia [5], and are found within a variety of mammalian definitive hosts, including raccoons (*Procyon lotor* Linnaeus, 1758), skunks (*Mephitis* spp. Schraber, 1776; *Conepatus* spp. Gray, 1837; and *Spilogale* spp. Linnaeus, 1758), badgers (*Taxidea taxus* Schreber, 1777), martens (*Martes americana* Scheber, 1778), fishers (*Martes pennati* Erxleben, 1777), marmots (*Marmota* spp. Blumenbach, 1779), and bears (*Ursus* spp. Linnaeus, 1758) [6]. Of the various *Baylisascaris* spp., *Baylisascaris procyonis*, the roundworm of raccoons, is considered the most pathogenic because of its aggressive larval migration in aberrant hosts [7]. Due to its pathogenicity, *B. procyonis* has been the most studied of the *Baylisascaris* spp. [2,6,8,9].

The *B. procyonis* life cycle begins when adult females produce eggs, which are shed in the feces of infected raccoons [6,10]. Juvenile raccoons become infected after ingesting larvated eggs (direct transmission), while adult raccoons acquire infection after ingesting paratenic hosts (indirect transmission) [2]. *Baylisascaris procyonis* adults are host specific, only using raccoons as definitive hosts. In contrast, larvae of *B. procyonis* are not host-specific, and over 150 species of animals including birds, rodents, lagomorphs, carnivores, and primates (including humans) have been documented as paratenic hosts [2,11].

Humans are exposed to infective *B. procyonis* eggs shed in raccoon feces. On a per day basis, raccoon feces have been documented to contain a mean of 16,563 ± 4321 *B. procyonis* eggs per gram of feces [12]. With a mean wet weight of 40.8 ± 13.7 g/raccoon feces [12], as raccoon feces decay, hundreds of thousands of *B. procyonis* eggs are deposited into the soil. Ogdee et al. [13] noted that depending upon the decay process (i.e., desiccation, precipitation, and/or wind), *B. procyonis* eggs can be found 28–68 cm from the defecation spot; hence, a single *B. procyonis*-infected raccoon fecal sample could potentially contaminate an area of 0.25–1.45 m^2^. Within the environment, up to 92% of *B. procyonis* eggs have been demonstrated to remain viable and thus infective for at least two years [14]. Additionally, due to their adhesive outer coating, 60% of the eggs remain on the soil surface [14]. Raccoon latrines can serve as the foci of parasitic transmission; thus, when raccoons live in close proximity to humans, risk of zoonotic transmission to humans increases [15]. For example, 51% of 119 backyards in suburban Chicago, Illinois, contained raccoon latrines, with 23% of the latrines containing *B. procyonis* eggs [15].

Historically and presently, *Toxocara canis* Werner, 1782 is considered the most important ascarid etiologic agent of visceral larva migrans (VLM) and ocular larva migrans (OLM) in humans [16], mainly because of the close relationship between humans and domestic dogs. However, *B. procyonis* is considered more pathogenic than *T. canis* and can cause neural larva migrans (NLM) [8]. In humans, the larvae of *B. procyonis* can cause fatal or severe neurologic disease by invading the spinal cord and brain (i.e., NLM), blindness by invading the eye (i.e., OLM), and mimic a multitude of illnesses by invading the viscera (i.e., VLM) [8]. To date, 25 *Baylisascaris* NLM encephalitis cases in humans from the United States and Canada have been documented, with additional cases occurring each year [3]. Even though *B. procyonis* has been well-documented in human cases from 2000 to 2015, *B. procyonis* is still an often-ignored parasite by human physicians in their lists of differential diagnoses [3]. Most human cases involve children who are at risk due to poor hygiene and oral sampling of their environment [17,18,19]; however, adults also are susceptible to severe NLM [3]. Additionally, about 30 cases of *Baylisascaris* OLM have occurred worldwide, mostly in adults where raccoons and skunks live synanthropically with humans [3].

The goal of this study was to demonstrate that any location with *B. procyonis* helminths, definitive hosts, and proximity to humans can create the potential health risk of baylisascariasis. Therefore, the objectives of this study were to (1) determine the level of soil contamination by *B. procyonis* eggs within a suburban area with a known population of raccoons, (2) determine if differences in soil contamination occurred between wild animal defecation sites and random soil samples within a suburban area, and (3) offer suggestions to reduce human risk of *B. procyonis* exposure.

## 2. Materials and Methods

### 2.1. Study Area

The Oso Creek area of southern Corpus Christi (27°39′06″ N, 97°24′25″ W, 7 m elevation), Nueces County, TX, USA, was selected as the study location to assess the potential risk of *B. procyonis* exposure. The area is newly developed (<25 years old), typical US suburbia with an average of ~350 households/km^2^ [20] that maintain landscaped yards (i.e., live oak (*Quercus virginiana* Miller, 1768) and honey mesquite (*Neltuma glandulosa* Torrey, 1845) are common trees), a known raccoon density of 2.1 ± 0.5 raccoons/ha [14], and an average of 3.7 people/household [21].

### 2.2. Experimental Design

An informational flyer about *B. procyonis* and its associated health risks was placed on the front doors of approximately 600 houses in the Oso Creek area. The flyer contained information about the helminth, potential mammalian paratenic hosts, how it is transmitted, potential risks to humans, and how to reduce one’s risk of exposure [22]. The flyer also described the current experiment and asked residents if they were willing to participate in the study, and if selected, they would have the soil of their property tested for *B. procyonis* eggs. In total, 463 (77%) property owners were willing to participate in this study; of these 334 owners (72%) stated that they had observed raccoons on their property or had raccoons defecate on their property. From these 334 respondents, 100 properties (30%) were randomly selected from which to collect soil samples.

Soil samples were collected from May to July 2021. Property owners identified sites where raccoons had been observed on their property. Soil columns were collected with a 2.7 cm diameter AMS soil probe (Forestry Suppliers, Jackson, MS, USA) from each site identified by the property owner where a raccoon or its feces was observed. A random soil sample from the property also was collected by locating the southwestern most corner of the house and walking 20 m due south and using this location as a starting point. A random number generator was used to select one of the eight cardinal directions with 1 = south, 2 = southeast, 3 = east, and proceed in a clockwise fashion until 8 = southwest. Once the direction was selected, a random number was selected from 1 to 10, the randomly selected number of meters was walked and a soil column was collected at that point as described above. Each soil sample was placed in an individualized plastic bag, which was labeled for property, date, and soil site within the property. Soil probe was thoroughly cleaned between sample collections.

At the laboratory, soil samples were held at room temperature (i.e., ~22 °C) for one month to allow time for eggs to larvate [9]. After which, bags containing eggs were processed using a centrifugal sedimentation-floatation method [23]. Briefly, soil was treated with a 20% bleach solution to remove the outer protein coat of any eggs, making them non-adherent [23]. Eggs, if present, from each soil sample were concentrated by centrifugation and counted with a Beckman Coulter cell counter (Z series, Beckman Coulter, Indianapolis, IN, USA). *Baylisascaris procyonis* were identified in wet mounts using features according to Bowman [24]. The two most common synanthropic definitive hosts within this area were raccoons, potentially carrying *B. procyonis,* and striped skunks (*Mephitis mephitis*), potentially carrying *B. columnaris* Leidy, 1856. However, property owners stated that striped skunks were uncommon in their neighborhoods. Thus, *B. procyonis* would be the most likely *Baylisascaris* spp. found within the area. A sample of eggs were placed on a hemocytometer and 100 *B. procyonis* eggs were counted to determine motility of larvae in eggs, using motility as a proxy for viability [25]. Soil samples that contained at least one viable, verifiable *B. procyonis* egg were considered positive.

### 2.3. Statistical Analysis

The chi-square goodness-of-fit analysis was used to determine if the frequencies of prevalence of *B. procyonis*-contaminated soil samples differed between randomly selected and owner-identified locations and if the frequency of positive *B. procyonis* locations differed between the locations identified by the property owners. All tests were considered significant at *p* < 0.05. All means are reported ± 1 standard error.

## 3. Results

One hundred random soil samples and an additional 141 soil samples from sites identified by the property owner as sites where raccoons or their scats were observed, were collected. Thirty-four (14.1%) of the soil samples contained *B. procyonis* eggs; these samples were from 27 of the 100 residential properties sampled (27%). Of the soil samples containing eggs, 12 (35.3%) were from randomly collected soil samples and 22 (64.7%) were from sites where the property owners had seen raccoons. Positive soil samples, on average, contained 31,287 ± 6943 *B. procyonis* eggs/gram of soil; on average, 92% (92.1 ± 2.3; x ± SE) of the *B. procyonis* eggs had motile larvae after 1 month.

One property had three sites that were positive for *B. procyonis* eggs; one randomly selected site and two sites identified by the property owner. A total of 6 properties had two sites identified by the property owner that were *B. procyonis* positive, and 10 properties had one *B. procyonis* positive site, which was identified by the property owner. Eleven other properties were positive for *B. procyonis* eggs at their randomly selected soil site.

Besides random sites within their yard, property owners identified woodpiles, tree bases, near porches, and the perimeter base of buildings (e.g., sheds, garages) as sites where raccoons were observed on their properties. However, the frequency of positive and negative *B. procyonis*-contaminated soil samples between randomly selected and owner-identified sites within the 100 sampled properties did not differ (*χ*^2^ = 0.62, df = 1, *p* = 0.43). In addition, the frequency of positive *B. procyonis* sites did not differ (*χ*^2^ = 6.29, df = 4, *p* = 0.18) between the sites identified by the property owners.

## 4. Discussion

This study demonstrates that *B. procyonis*-infected raccoons that live synanthropically with humans could expose humans to *Baylisascaris* eggs; and thus, those humans have a significant health risk to this potentially devastating parasite. The majority of those who are exposed to *B. procyonis* are unaware of their exposure until their health has deteriorated due to NLM or OLM and they have experienced central nervous system (CNS) impairment, seizures, blindness, or cognitive impairment [3]. However, exposure to *B. procyonis* is likely higher than the number of documented infections with this parasite because there is a difference between exposure (i.e., coming into contact with eggs), infection (i.e., ingestion of eggs that hatch into larvae, which migrate in the body), and development of baylisascariasis (i.e., progression of infection, which lead to health complications) [3]. Routine serological testing by physicians for this parasite among humans is uncommon; however, when serological testing occurred, subclinical infections were discovered [26].

The suburban neighborhood in Corpus Christi, Texas, was used as a case study to demonstrate that exposure to *B. procyonis* is possible to occur wherever the parasite, definitive hosts, and humans co-occur. Once considered a health threat specific to the midwestern United States (i.e., Indiana, Michigan, Illinois, and Ohio), *B. procyonis* in raccoons has now been documented throughout nearly the entire 48 contiguous states [3].

The potential exposure to humans is exacerbated by the fact that one *B. procyonis*-infected raccoon scat can contain >500,000 *B. procyonis* eggs [12], which upon decay, by desiccation or precipitation, could contaminate up to a 1-m^2^ area [13]. Therefore, a single *B. procyonis*-infected raccoon can contaminate 0.03 ± 0.1 ha/yr with *B. procyonis* eggs [13], with 60% of those eggs staying on the soil surface and remaining viable for at least 2 years [14]. In addition, raccoons use latrine sites for defecation and a single raccoon, on average, visits as many as six latrine sites during a 2-week period [27]. The use of multiple latrine sites by raccoons can cause higher rates of contamination of *B. procyonis* at such sites than the infection rate found in raccoon populations or in individual raccoon fecal samples [27]. If a human is exposed to such a highly contaminated area, they could potentially acquire a large enough dose of *B. procyonis* eggs to develop CNS disorders. Tiner [28] noted that only 5−7% of *B. procyonis* larvae that were ingested by rodents need to enter the brain to result in clinical disease.

To date, treatment for patients with CNS disorders caused by *B. procyonis* is the larvicidal drug albendazole in combination with steroids to reduce inflammation [6]. Early treatment, if possible, is best to kill migrating larvae and limit further damage. However, in most reported cases, treatment was ineffective and CNS disease progressed [3]. The ovicidal effect of three saprophytic fungi were tested on *B. procyonis* eggs as a means of treatment for *B. procyonis*-contaminated sites, but the addition of fungi only reduced egg viability by 50–66%, on average [29].

Nearly 100% of the property owners who received the flyer describing this study and the risks posed by *B. procyonis* had never heard of this parasite and were unaware that it constituted a potential health risk. The general public and potentially physicians are unaware of the inherent dangers of this zoonotic parasite [30]. With the global spread of raccoon introductions, educating the public about the health risks of *B. procyonis* is critical. Raccoons now can be found in southern Canada [3], Mexico (except Baja California), and Central America [3], throughout most of Europe (i.e., Spain, France, Poland, Slovakia, Ukraine, Belarus, Estonia, Lithuania, Germany, Belgium, Denmark, The Netherlands, England, Norway, Sweden, and Italy [31,32]), and into Asia (i.e., Russia, Japan, and China; [33,34,35]). Unfortunately, there appears little discussion concerning the inherent dangers of *B. procyonis* in advance of raccoon translocations. For example, Al-Sabi et al. [36] noted that zoo personnel and local veterinarians who supervised captive raccoons within Denmark zoos displayed little knowledge of *B. procyonis* and its zoonotic potential. Therefore, greater worldwide public education efforts concerning *B. procyonis*, especially in areas with high raccoon densities, are warranted.

Although this study focused on the potential health risks of *B. procyonis* to humans created by raccoons, there are other species of *Baylisascaris*, each with their own definitive host. For example, striped skunks, which may be infected with *B. columnaris*, are also synanthropic with humans and could contaminate suburban environments with *B. columnaris* eggs. In addition, *B. melis* Gedoeist, 1920 occurs in badgers, *B. devosi* Sprent, 1952 in martens and fishers, *B. laevis* Leidy, 1856 in marmots and ground squirrels, *B. transfuga* Rudolphi, 1819 in bears, *B. venezuelensis* Mata et al., 2016 in South American spectacled (Andean) bears (*Tremarctos ortnatus* Cuvier, 1825), and *B. potosis* Tokiwa et al., 2014 in kinkajous (*Potos flavus* Saint-Hilaire and Cuvier, 1795) [2,37]. Although too few studies have been conducted concerning the potential health risk of the other *Baylisascaris* species to humans [37], that fact does not reduce those species’ potential exposure risks to humans. Until the pathogenicity of each *Baylisascaris* spp. is known, caution is advisable to reduce exposure to fecal matter of potential definitive hosts of any *Baylisascaris* spp.

Unregulated trade and illegal markets in wildlife species exacerbate the potential spread of zoonotic diseases and parasites [38], of which *Baylisascaris* spp. can be spread worldwide due to the large number of susceptible mammalian species as definitive and paratenic hosts [3]. Thus, baylisascariasis should be considered a global problem. Physicians need to include baylisascariasis within their list of differential diagnoses when the presentation of clinical signs of meningoencephalitis and ocular larva migrans occur.

## 5. Conclusions

Although this study was conducted in just one suburban location, it is offered as a case study that can be applied globally wherever hosts of *Baylisascaris* spp. occur. As habitat fragmentation due to urbanization and suburban sprawl continue to expand into wildlife habitat, there is an increased likelihood of human–wildlife interactions [39]. One such interaction is the potential of exposure to parasitic diseases, as demonstrated by this study. The public needs to be informed of the potential health dangers of synanthropic wildlife species and to be educated as to how to reduce the likelihood of exposure to such diseases.

The best method to combat baylisascariasis is to take precautions to reduce exposure to *B. procyonis* eggs. Below is a list of recommendations to reduce exposure specifically to *B. procyonis* and raccoons; however, most suggestions are pertinent to most definitive and paratenic hosts of *Baylisascaris* spp.

1. Raccoons are not pets. They are wild animals and should remain as such. Numerous adult humans who were diagnosed with OLM or subacute neuroetinitis admitted to having a pet raccoon [3].
2. Eliminate enticements that attract raccoons to your property. Enticements include, but are not limited to, self-pet feeders, pet doors, brush piles, and fallen fruit from trees. As fruit ripens on trees, the fruit will fall to the ground, creating a food source that could easily be exploited by multiple raccoons. It is prudent to discard fallen and unwanted fruit from your property. Although self-pet feeders are convenient, they provide an easy source of food for raccoons as well.
3. When playing and working outdoors, it is pragmatic to wear shoes and gloves. If being barefoot is a necessity for outdoor enjoyment, thoroughly wash hands and feet when returning indoors, and especially before eating.
4. Vigilantly watch toddlers as they have a propensity to place items in their mouth when playing outdoors; such a behavior should be discouraged. If children have sandboxes in which to play and dig, invest in a tight cover to keep animals from using the sandbox as a litter box.
5. Trim tree branches away from your house and secure any open or damaged areas of your home. Raccoons can find even small openings as access points to enter attics, basements, etc.
6. If you have a home-grown garden for vegetables and fruits, thoroughly wash your produce before consuming it.
7. If you are aware of a raccoon latrine site, extreme heat (>62 °C) is the best method to kill *B. procyonis* eggs [25]. Direct flame from a hand-held propane torch has been demonstrated as effective to kill *B. procyonis* eggs [40]. Flame the scat until ash and flame the soil for >30 s, sift the soil with a rake, and then flame again for another 30 s to increase the likelihood of killing the eggs [40]. However, check local laws and city ordinances regarding the use of fire devices within neighborhoods prior to use.

It may be impossible to eliminate 100% of the potential risk, but with diligence, the risk of exposure to this zoonotic parasite can be reduced. Efforts to inform local health care professionals, public health officials, veterinarians, and the general public about the human health and safety risks associated with *Baylisascaris* spp. should be made.

## Data Availability

Corresponding author will make data available upon request.
